# Syndrome de résistance à l’Adrénocorticotrophine Hormone (ACTH): à propos d’un cas

**DOI:** 10.11604/pamj.2018.30.244.15541

**Published:** 2018-08-02

**Authors:** Morgiane Solange Tognidé Sêlomin Houngbadji, Babacar Niang, Djibril Boiro, Aminata Mbaye, Abdoulaye Seck, Abdoulaye Aliou Ndongo, Indou Deme Ly, Ousmane Ndiaye

**Affiliations:** 1 Centre Hospitalier National d’Enfants Albert Royer-Fann, Dakar, Sénégal; 2 Service de Pédiatrie du Centre Hospitalier Abass Ndao de Dakar, Dakar, Sénégal; 3 Institut Pasteur de Dakar, Dakar, Sénégal; 4 Hôpital Aristide Le Dantec, CHU de Dakar, Dakar, Sénégal

**Keywords:** Résistance à l´ACTH, insuffisance cortisolique, hypoglycémie, ACTH resistance, cortisol deficiency, hypoglycaemia

## Abstract

Le syndrome de résistance à l'Adrénocorticotrophine Hormone (ACTH) est l'une des rares causes d'insuffisance surrénalienne chez l'enfant. Toutes les formes d'insensibilité héréditaire à l'ACTH décrites à ce jour sont d'origine autosomique récessive. Dans nos pays à ressources limitées, bon nombre de ces pathologies rares sont méconnues ou non diagnostiquées, en raison des plateaux techniques insuffisants. Nous rapportons l'observation d'un nourrisson de 4 mois hospitalisé pour des hypoglycémies réfractaires et chez qui; malgré la présence d'une mélanodermie généralisée et importante, des troubles digestifs, et des troubles ioniques, le diagnostic d'insuffisance cortisolique n'a été évoqué que rétrospectivement au décours d'un arrêt respiratoire avec une évolution favorable et dépendante de l'hydrocortisone. L'objectif de ce travail est donc, de mettre en exergue les particularités, cliniques, biologiques et thérapeutiques des déficits périphériques en cortisol, en dehors des blocs enzymatiques, notamment ce syndrome de résistance à l'ACTH.

## Abstract (en)

Adrenocorticotropic hormone (ACTH) insensitivity syndrome is one of the rare causes of adrenal insufficiency in children. All described inherited ACTH insensitivity forms are of autosomal recessive origin. In our resource-poor Countries, many of these rare diseases are ignored or not diagnosed due to inadequate technical equipments. We report the case of a 4-month old infant hospitalized for refractory hypoglycaemias. Despite the patient had generalized and severe melanodermia, digestive disorders and ion channel disorders the diagnosis of cortisol deficiency was only diagnosed retrospectively during respiratory arrest with favorable outcome under hydrocortisone therapy. This study aims to highlight the clinical, laboratory and therapeutic features of peripheral cortisol deficiency, without enzymatic blocks, including this adrenocorticotropic hormone (ACTH) insensitivity syndrome.

## Introduction

Les syndromes de résistance à l'ACTH ou insensibilité à l'ACTH constituent un ensemble de maladies à incidence faible dont il existe au moins trois formes moléculaires différentes: le syndrome de déficit familial isolé en glucocorticoïdes (*familial glucocorticoid deficiency* (FGD)) qui comprend deux sous types (FGD 1 et FGD 2) et le syndrome des 3A (ACTH résistance, Achalasie, Alacrimie) [1]. Ils représentent l'une des rares étiologies de l'insuffisance surrénalienne chez l'enfant et sont caractérisées de façon générale par un déficit sévère en glucocorticoïdes. La forme la plus simple est le déficit familial isolé en glucocorticoïdes (FDG) avec toute une cohorte de symptômes associés à ce déficit [1]. Elle a une prévalence de 1/14.000 naissances [2]. Les manifestations cliniques sont précoces, néonatales ou infantiles, et caractérisées par des hypoglycémies répétées. Le retard diagnostique est fréquent parce qu'on y pense pas suffisamment, et dans nos pays à ressources limitées, ce retard est davantage marqué par le plateau technique insuffisant. Nous rapportons l'observation d'un nourrisson de 4 mois au CHU de Dakar, atteint de ce rare syndrome et dont le pronostic vital a été engagé par ses symptômes graves.

## Patient et observation

**Figure 1:**
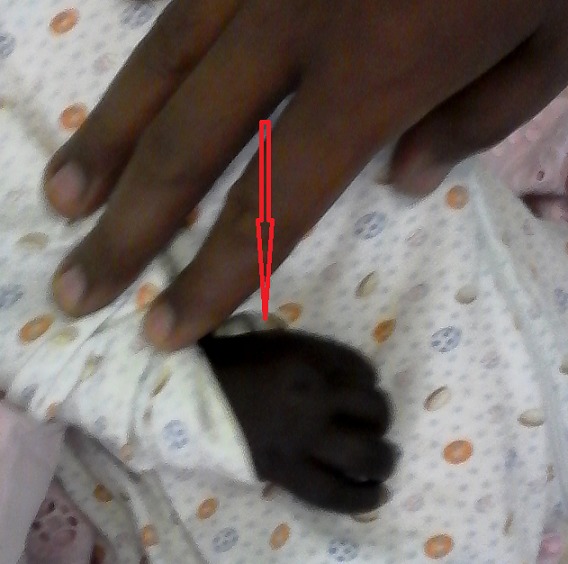
Mélanodermie de la main

**Figure 2:**
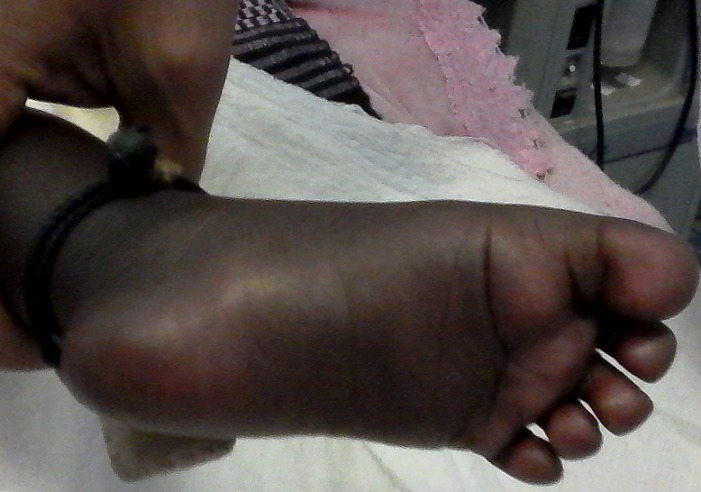
Mélanodermie plante du pied

A.D est un nourrisson de 4 mois de sexe féminin admise pour convulsions non fébriles. L'interrogatoire avait retrouvé des vomissements incoercibles et une diarrhée évoluant depuis 3 jours. Ce nourrisson avait des antécédents d'asphyxie périnatale compliquée d'encéphalopathie anoxo ischémique stade 2 de Sarnat associée à une infection néonatale, et son père était épileptique. Il avait également une consanguinité parentale au 1^er^degré. L'examen physique à l'admission avait objectivé un ictère conjonctival franc, une hypoglycémie sévère à 0,31g/L, une hypothermie à 33,6°C, une détresse respiratoire. On notait aussi une mélanodermie généralisée plus marquée aux dos et dans la paume des mains, la plante des pieds, les lèvres, la face interne des joues et les gencives (Figure 1, Figure 2). Il n'y avait pas d'anomalies de la différenciation sexuelle; de même le reste de l'examen physique était normal. L'ionogramme sanguin fait en urgence retrouvait une hyponatrémie sévère à 117 mmol/L; une hyperkaliémie à 5,7 mmol/L. La calcémie était normale, de même que la magnésémie et la phosphorémie. Ailleurs un bilan infectieux fait, était négatif. Ce tableau clinique s'aggravait malgré un traitement symptomatique adéquat (apports glucidiques de 8 mg/kg/min) avec des hypoglycémies profondes (0,16g/L) et réfractaires, l'apparition d'une hépatomégalie, puis un arrêt respiratoire. Le bilan biologique réalisé après les mesures de réanimation montrait une cholestase hépatique avec hyperbilirubinémie à prédominance conjuguée (bilirubine totale: 105,7 mg/L et bilirubine directe: 71,37 mg/L), une élévation des phosphatases alcalines (1074UI/L) et des transaminases (ASAT/ 162 UI/L, ALAT / 148 UI/L). Devant le caractère réfractaire de l'hypoglycémie, un traitement à base d'hydrocortisone (dose de charge de 100 mg/m^2^/jour et doses d'entretien à 15 mg/ m^2^/jour) a été introduit sans hormonologie préalable, celle-ci n'étant pas disponible en urgence. L'évolution ultérieure était favorable et dépendante de l'hydrocortisone au bout de 72 heures (régression de l'ictère et de l'hépatomégalie, normalisation de l'état respiratoire et neurologique de même que la natrémie et la kaliémie). Le diagnostic d'une insuffisance surrénalienne a donc été évoqué rétrospectivement et confirmé par la cortisolémie de 8 heures qui était nulle. Dans le cadre de la recherche étiologique un test de stimulation au synacthène retard avait été fait et montré des cortisolémies à H0 et H6 (6 heures) nulles (0 ng/mL) et une ACTHmie très élevée supérieure à 2.000 ng/mL soit plus de 30 fois la normale. Le Tableau 1 résume le bilan sanguin hormonal réalisé. L'examen morphologique des surrénales (échographie et TDM abdominales) était normal. Par ailleurs, une ectopie rénale gauche était notée. Ce tableau d'insuffisance surrénalienne périphérique avec déficit isolé en glucocorticoïdes sans blocs enzymatiques, sans anomalies morphologiques des surrénales nous a fait évoquer un syndrome de résistance à l'ACTH, bien que nous ne disposons pas de l'étude génétique, faute d'un plateau technique adéquat. L'évolution ultérieure sous hydrocortisone per os à 10 mg/m^2^/jour est favorable avec une bonne croissance staturo pondérale.

**Tableau 1:** Explorations hormonales réalisées

Hormones	Valeurs observées	Valeurs normales
Cortisol	0 ng/Ml	37-194 ng/L
ACTH	2000 ng/mL	5-60 ng/L
Aldostérone	219 pg/mL	20-1100 pg/mL
rénine active	169,90 pg/mL	˂ 20 pg/mL
delta 4 – androstènedione	˂ 0,05 ng/mL	0,05-0,15 ng/mL
17-hydroxy prégnénolone	˂ 0,10 ng/mL	0,10-0,5 ng/mL
17 alpha hydroxy progestérone	˂ 0,10 ng/mL	˂ 0,05-0,6 ng/mL
11 désoxycortisol	0,10 ng/mL	0,10-1,00 ng/mL

## Discussion

Les syndromes de résistance à l'hormone adrénocorticotrophique parmi lesquelles, on retrouve le syndrome de déficit familial isolé en glucocorticoïdes (*familial glucocorticoid deficiency* (FGD)) et le syndrome des 3A appartiennent à un groupe de désordres rares à transmission autosomique récessive et caractérisés par une insensibilité à l'ACTH [3]. L'ACTH stimule la synthèse corticosurrénalienne de cortisol et d'androgènes via le récepteur à l'ACTH. Ce dernier, encore appelé récepteur mélanocortine de type 2 (MC2R), est un récepteur à sept domaines transmembranaires couplé aux protéines G appartenant à la famille des récepteurs de la mélanocortine [3]. Des mutations inactivatrices de ce récepteur ont été mises en évidence dans certains cas de FGD. Il s'agit des FGD de type 1 qui ne représentent que 25 % des FGD [4, 5]. Les autres cas de FGD sont de pathogénie plus obscure. L'hypothèse de l'implication de cofacteurs indispensables à l'expression de MC2R a conduit à la découverte de mutations affectant une protéine accessoire du récepteur de la mélanocortine dénommée *melanocortin receptor accessory protein*. Ces mutations sont responsables d'environ 20 % des FGD dits de type 2 [4, 6]. Le FGD peut être isolé; il se révèle alors dans la période néonatale ou durant les 3 premières années de vie, et seule la sécrétion de glucocorticoïdes est atteinte [3]. C'est le cas de notre patiente, issue d'un mariage consanguin, dont la symptomatologie a été exprimée à l'âge de 4 mois et dont la biologie a confirmé ce déficit isolé en glucocorticoïdes. Elle n'a jamais présenté de syndrome de perte de sel en dehors de l'épisode de décompensation aigue au cours duquel le diagnostic d'insuffisance surrénalienne a été posé. Dans la grande majorité des cas (82%), les patients présentent des crises d'hypoglycémie plus ou moins sévères, et pouvant entraîner des pertes de connaissance avec sueurs, des vomissements, des convulsions, voire un coma qui peut être fatal [1]. Notre patiente aussi, avait présenté ces hypoglycémies profondes ayant entrainé chez elle la conséquence la plus dramatique qu'est l'arrêt respiratoire. En plus des hypoglycémies, notre patiente présentait un syndrome de cholestase franc. Kershnar AK *et al* soulignent le fait que ces patients peuvent présenter un teint jaune dû à une forme transitoire d'hépatite dépendante des glucocorticoïdes [7]. Elle présentait également cette hyperpigmentation généralisée et très marquée aux dos des mains et des gencives comme rapporté dans la littérature, où elle apparaît dans 91% des cas dès le premier mois de vie, mais surtout après 3 à 5 mois [7-9].

Mais malgré toute cette symptomatologie clinique typique, chez notre patiente, le diagnostic de résistance à l'ACTH n'a pu être posé que 8 mois plus tard étant donné la rareté du syndrome (1/14.000 naissances) et le peu de littérature disponible faisant que le praticien n'y pense pas souvent [**2**]. En effet, les principales étiologies de l'insuffisance surrénalienne de l'enfant sont représentées par les blocs enzymatiques, dont le déficit en 21 hydroxylase au devant de la scène, avec un trouble de la différenciation sexuelle chez la fille. Au plan biochimique, les patients atteints de FGD ont des taux plasmatiques de cortisol (prélèvement réalisé entre 8 et 9 heures) toujours faibles à indétectables (< 20 nmol/l) alors qu'ils sont en situation de stress important. De façon occasionnelle, les taux rencontrés sont à la limite inférieure. Les taux urinaires de 17- hydroxycorticostéroïdes sont également effondrés. Les taux plasmatiques d'ACTH sont au contraire très élevés: des valeurs supérieures à 1.000 ng/l sont souvent rencontrées. L'administration intraveineuse de synacthène (250µg/ m^2^ de surface corporelle et mesurer à 0, 30 et 60 minutes), ne permet pas une augmentation significative des taux de cortisol chez la plupart des patients [5]. Ces particularités biochimiques ont été observées chez notre patiente ; ses cortisolémies à H0 et H6 (6 heures) après stimulation au synacthène retard étaient nulles et son taux plasmatique d'ACTH > 2000 ng/l. Par contre, dans tous les cas rapportés, les taux plasmatiques d'aldostérone sont normaux avec juste quelques perturbations mineures dans certains cas [5]. Notre patiente n'a pas dérogé à cette règle. A ces valeurs normales sont associés des niveaux plasmatiques normaux d'électrolytes comme chez notre patiente en dehors des épisodes de décompensation aigue ; il n'y a pas de perte de sel. Le traitement est relativement simple dans la mesure où seule une substitution glucocorticoïde est nécessaire par un traitement oral à l'hydrocortisone. De façon occasionnelle on lui préfère un traitement à la dexaméthasone qui présente l'avantage d'avoir une action plus longue. Le traitement doit être ajusté pour éviter l'apparition de troubles liés à l'hypercortisolisme. Un traitement substitutif minéralocorticoides n'est pas nécessaire [1]. A.D est sous hydrocortisone depuis son diagnostic et son évolution est favorable. L'ensemble de ces caractéristiques, à la fois cliniques et biochimiques, indique clairement un déficit isolé en glucocorticoïdes avec une fonction rénine-angiotensine préservée, due à une insensibilité à l'ACTH circulant.

## Conclusion

Notre observation souligne l'importance de la considération sémiologique clinique des pathologies en dépit de leur fréquence (rare ou non) et une démarche diagnostique étiologique rigoureuse en présence d'hypoglycémie. Les caractéristiques sémiologiques du déficit cortisolique pur en période néonatale ou chez le petit nourrisson (ictère cytolytique et hypoglycémie) doivent être connues de tous les pédiatres pour un diagnostic et une prise en charge précoce avant une décompensation hémodynamique pouvant être fatale.

## Conflits d’intérêts

Les auteurs ne déclarent aucun conflit d'intérêts.
